# Different factors associated with loss to follow-up of infants born to HIV-infected or uninfected mothers: observations from the ANRS 12140-PEDIACAM study in Cameroon

**DOI:** 10.1186/s12889-015-1555-2

**Published:** 2015-03-07

**Authors:** Larissa Kamgue Sidze, Albert Faye, Suzie Ndiang Tetang, Ida Penda, Georgette Guemkam, Francis Ndongo Ateba, Jean Audrey Ndongo, Félicité Nguefack, Gaëtan Texier, Patrice Tchendjou, Anfumbom Kfutwah, Josiane Warszawski, Mathurin Cyrille Tejiokem

**Affiliations:** Service d’Epidémiologie et de Santé Publique, Centre Pasteur du Cameroun, Membre du Réseau International des Instituts Pasteur, Yaoundé, Cameroun; ISPED, Université Victor Segalen, Bordeaux II, France; Assistance Publique des Hôpitaux de Paris, Pédiatrie Générale, Hôpital Robert Debré, Paris, France; Université Paris 7 Denis Diderot, Paris Sorbonne Cité, Paris, France; Service de Pédiatrie, Centre Hospitalier d’Essos, Yaoundé, Cameroun; Hôpital de Jour, Hôpital Laquintinie, Douala, Cameroun; Faculté de Médecine et des Sciences Pharmaceutiques, Université de Douala, Douala, Cameroun; Centre Mère et Enfant de la Fondation Chantal Biya, Yaoundé, Cameroun; Faculté de Médecine et des Sciences Biomédicales, Université de Yaoundé I, Yaoundé, Cameroun; SESSTIM (UMR 912), Université Aix-Marseille, Marseille, France; Service de Virologie, Centre Pasteur du Cameroun, Membre du Réseau International des Instituts Pasteur, Yaoundé, Cameroun; Equipe 4 (VIH et IST) - INSERM U1018 (CESP), Le Kremlin Bicêtre, Paris, France; Assistance Publique des Hôpitaux de Paris, Service d’Epidémiologie et de Santé Publique, Hôpital de Bicêtre, Le Kremlin Bicêtre, Paris, France; Université de Paris Sud 11, Paris, France

**Keywords:** Loss to follow-up, Failed to return for scheduled clinical visits, Associated factors, Cohorts of HIV-exposed and -unexposed infants

## Abstract

**Background:**

Loss to follow-up (LTFU) is a cause of potential bias in clinical studies. Differing LTFU between study groups may affect internal validity and generalizability of the results. Understanding reasons for LTFU could help improve follow-up in clinical studies and thereby contribute to goals for prevention, treatment, or research being achieved. We explored factors associated with LTFU of mother-child pairs after inclusion in the ANRS 12140-Pediacam study.

**Methods:**

From November 2007 to October 2010, 4104 infants including 2053 born to HIV-infected mothers and 2051 born to HIV-uninfected mothers matched individually on gender and study site were enrolled during the first week of life in three referral hospitals in Cameroon and scheduled for visits at 6, 10 and 14 weeks of age. Visits were designated 1, 2 and 3, in chronological order, irrespective of the child’s age at the time of the visit. Mother-child pairs were considered lost to follow-up if they never returned for a clinical visit within the first six months after inclusion. Uni- and multivariable logistic regression were adjusted on matching variables to identify factors associated with LTFU according to maternal HIV status.

**Results:**

LTFU among HIV-unexposed infants was four times higher than among HIV-exposed infants (36.7% vs 9.8%, p < 0.001). Emergency caesarean section (adjusted Odds Ratio (aOR) = 2.46 95% Confidence Interval (CI) [1.47-4.13]), young maternal age (aOR = 2.29, 95% CI [1.18-4.46]), and absence of antiretroviral treatment for prophylaxis (aOR = 3.45, 95% CI [2.30-5.19]) were independently associated with LTFU among HIV-exposed infants. Factors associated with LTFU among HIV-unexposed infants included young maternal age (aOR = 1.96, 95% CI [1.36-2.81]), low maternal education level (aOR = 2.77, 95% CI [1.95-3.95]) and housewife/unemployed mothers (aOR = 1.56, 95% CI [1.16-2.11]).

**Conclusion:**

Failure to return for at least one scheduled clinical visit is a problem especially among HIV-unexposed infants included in studies involving HIV-exposed infants. Factors associated with this type of LTFU included maternal characteristics, socio-economic status, quality of antenatal care and obstetrical context of delivery. Enhanced counselling in antenatal and intrapartum services is required for mothers at high risk of failure to return for follow-up visits.

**Electronic supplementary material:**

The online version of this article (doi:10.1186/s12889-015-1555-2) contains supplementary material, which is available to authorized users.

## Background

HIV/AIDS remains a disease of public health importance and mother-to-child transmission (MTCT) is one of the major problems [1]. Recent estimates of the Joint United Nations Program on HIV/AIDS (UNAIDS) indicate that approximately 330,000 children worldwide under 15 years old became infected with HIV in 2012. Sub-Saharan Africa is the most severely affected region, accounting for more than 90 percent of paediatric HIV infections [1]. Most of these infections occurred during pregnancy, delivery or breastfeeding making the prevention of mother-to-child transmission (PMTCT) a public health priority [2]. Over the last few years, efforts have been made in Sub-Saharan countries to improve PMTCT [3-5]. However, the effectiveness and efficacy of these interventions are profoundly affected by high rates of loss to follow-up (LTFU) [6]. Studies in Malawi, Ivory coast and Angola have reported cumulative losses in PMTCT programs of 20-28% during antenatal care, to 70% four months post-partum and close to 81% six months after birth [7-9]. LTFU has major implications for the understanding of the true mortality rates among both HIV-positive and HIV-exposed children, as well as for accurate determinations of HIV transmission rates from mother to child [10].

Poor socioeconomic conditions have been identified as the main reason for LTFU in such studies and PMTCT programs. They include poverty, lack of paternal support, poor mobility, long distances between residence and care structures, and the cost of transport [11]. Therefore, it is possible that in a context of favourable conditions (free medical support, reminder phone calls, and incentives including food vouchers or reimbursement of transport fares) for follow-up, the problem of LTFU would be overcome. LTFU is a problem not only for PMTCT programs but also for clinical trials. LTFU in clinical trials can compromise study findings by reducing the power of a study to detect a true difference between the control and the intervention group. Differential LTFU may lead to bias through exaggerated effects in one of the groups. These issues can affect the results of a trial and in particular its generalizability and internal validity [12]. Understanding the reasons for LTFU among mother-infant pairs therefore could improve follow-up in clinical studies; this in turn may help achieve goals for prevention, treatment, or research. The main objective of this study was to determine factors associated with the failure of mother-child pairs to return for scheduled clinical visit (even once) in the ANRS 12140-Pediacam study in Cameroon.

## Methods

### Data source

Data used in this analysis were obtained from the ANRS-Pediacam cohort based in three referral hospitals in Cameroon (The Maternity of the Central hospital/Mother and Child Center of the Chantal Biya Foundation (MCH/MCC-CBF), Essos Hospital Center in Yaoundé (EHC) and the Laquintinie Hospital in Douala (LH)) and coordinated by the Centre Pasteur of Cameroon. The ANRS 12140-Pediacam study was designed to assess the feasibility of early diagnosis of HIV and early antiretroviral multitherapy in HIV-infected infants, and to evaluate the humoral response of these children to vaccines of the Expanded Program on Immunization (EPI).

The ANRS 12140-Pediacam is an ongoing prospective cohort study involving two consecutive phases. The first phase of the study included all infants born live to HIV-infected mothers with documented serostatus (group 1) identified before the 8th day of life from November 2007 to October 2010 and an equivalent number of infants born to HIV-uninfected mothers matched individually on gender and recruitment site. All newborns were expected to attend a clinical visit, according to the Cameroon National EPI calendar, at ages 6, 10 and 14 weeks for routine vaccination. Infant follow-up in the first phase was scheduled to coincide with this EPI timetable to minimise the number of visits to the hospital required. However, visits (follow-up visits) are designated hereafter as the first, second and third, independent of the age of the child. Samples for HIV virological testing were collected from HIV-exposed infants at the first follow-up visit (scheduled for 6 weeks), as previously described [13,14]. HIV test results were provided at the second visit (scheduled for 10 weeks). During the second visit, all parents/caregivers who received a negative result for their infants were counselled about how to avoid practises favouring HIV transmission to their infant. For breastfed infants whose first HIV test was negative were retested if they became symptomatic or six weeks after weaning if asymptomatic. For infants with a positive or indeterminate result from the first test, a blood sample was collected at the second visit for confirmatory testing. The results were announced at the third clinical visit (scheduled for 14 weeks).

Incentives, including free medical support for consultation, biological analysis, additional vaccines and reimbursement of transport costs, were provided to parents/caregivers by the project during follow-up visits.

All HIV-infected children and control groups of HIV-uninfected infants followed since birth, born to either HIV-infected or non-infected mothers were eligible for the second phase of the Pediacam project planned from 14 weeks to 5 years. This phase is not described here as it is not relevant to this study.

### Data collection

Socioeconomic and demographic characteristics of the families and obstetrical characteristics were collected at enrolment by questionnaire-based interview with mothers and examination of their hospital booklets. Questionnaires were also used subsequently to collect data on infant vital status, pathologies, vaccinations and HIV testing process as appropriate. Reminder phone calls were made to families who did not return for a follow-up visit within one week of the planned date.

### Ethical considerations

The ANRS 12140- Pediacam study has received ethical approval in Cameroon from the National Ethic Committee and in France from the Biomedical Research Committee of the Pasteur Institute of Paris. An administrative authorization was also delivered by the Cameroonian Ministry of Public Health. Written informed consent was obtained from parents or guardians prior to inclusion of infants into the research project.

### Study population

All infants born live to HIV-infected mothers and infants born to HIV-uninfected mothers matched on gender and recruitment site, enrolled from November 2007 to October 2010 into the first phase of the ANRS-Pediacam study planned from the first to 14^th^ week of life were eligible for this analysis.

### Main outcome definition and covariables

The main outcome was loss to follow-up (LTFU) or “failed to return for at least one scheduled clinical visit” defined as mother-child pairs who never returned for a clinical visit during the first 6 months of age after inclusion in the ANRS-Pediacam study. Those who attended clinical visits, even if only once, were not considered to be lost to follow-up. The threshold of six months was chosen because several studies indicate that knowing the HIV status of the child before age 6 months favours early initiation of HAART [15,16]. Returning for a clinical visit at least once within six months was perceived as having the willingness to comply with the study schedule; failure to attend subsequently may indicate loss of motivation due to constraints associated with the health system.

The covariables considered included variables pertaining to infant’s characteristics at birth (gender, prematurity, birth weight, APGAR, hospitalization at birth), maternal characteristics and socio-economic status (HIV serological status, marital status, level of education, socio-professional activity, monthly income, presence of a functional fridge at home, access to electricity, access to tap water), quality of antenatal care and obstetrical context of delivery (primigravid, mode of delivery, place of birth, number of antenatal visits, ART prophylaxis for PMTCT and disclosure of HIV status) and paternal characteristics (age, level of education and socio-professional activity).

### Statistical analyses

Maternal and infant characteristics are described using frequencies for categorical variables, medians and interquartile ranges for continuous variables. Their relation to LTFU was evaluated in each group defined by maternal HIV serostatus because of the differences observed between the two groups. For multiple births, only the first infant was included in the analysis. To examine covariables associated with LTFU, logistic regression models were adjusted on site and gender (matching variables) as appropriate for the matched study design [17]. The initial multivariable logistic regression model included those non collinear covariables found by univariable analysis to be associated with LTFU (as the dependent variable) with a p-value ≤ 0.25. The final model was obtained by successively removing variables not associated at a p-value <0.05 only if the odds ratios for the remaining variables were unchanged and taking interactions into account. The following known risk factors were maintained in the final model: low birth weight, prematurity and maternal educational level and socio-professional activity (as a surrogate for economic status). We performed a sensitivity analysis where we imputed missing data as a category for each variable. All statistical analyses were performed using R 2.15 software.

## Results

### Baseline characteristics of the study population

In total, 4104 mother-child pairs were enrolled in the first phase of the Pediacam study between November 2007 and December 2010, including 2051 HIV-exposed infants and 2053 HIV-unexposed infants. Among the 4104 mother-infant pairs initially enrolled, 68 were excluded because data for mother and infant characteristics at enrolment were not available. Further, 123 multiple gestation births were excluded to avoid duplication of maternal characteristics. Our analyses were thus conducted on the remaining 3913 mother-infant pairs (1964 HIV-exposed and 1949 HIV-unexposed). About 85.4% (3342/3913) of all the infants enrolled were born in the three maternities participating in the study, 13.9% (n = 542) in other maternities in the same cities and 0.7% (n = 29) at home or on their way to a healthcare facility. The two groups of infants differed significantly at enrolment (Table 1). The median maternal age at delivery was 28.6 years [IQR 24.8-32.5], significantly higher for HIV-infected mothers than in HIV-uninfected mothers (29.3 years vs 27.7 years, p < .001). A larger proportion of HIV-infected mothers than HIV-uninfected mothers delivered outside the Pediacam study sites (25.5% vs 3.6%, p < .001), were multiparous (84.6% vs 70.1%, p < .001) and had attended more than four antenatal visits (14.9% vs 9.2%, p < .001). Preterm birth (13.4% vs 10.6%, p = 0.009) and low birth weight infants (9.9% vs 5.8%, p < .001) were more frequent for HIV-infected than HIV-uninfected mothers.

**Table 1:** **Baseline characteristics of the study population in each group defined by maternal HIV serostatus, ANRS 12140- Pediacam study, Cameroon, 2007-2010**

**Characteristics (n)**	**Total**	**Maternal HIV serostatus**		**P value**
	**N = 3913**	**Positive (n = 1964)**	**Negative (n = 1949)**	
	**N (%)**	**n (%)**	**n (%)**	
**Mother’s age at delivery (years) (n = 3909)**				<0.001
<25	1023 (26.2)	385 (19.6)	638 (32.8)	
25-35	2417 (61.8)	1320 (67.2)	1097 (56.4)	
>35	469 (12.0)	258 (13.1)	211 (10.8)	
**Marital status (n = 3883)**				0.35
single/divorced/widowed	1173 (30.2)	604 (30.9)	569 (29.5)	
Married/living with a partner	2710 (69.8)	1351 (69.1)	1359 (70.5)	
**Maternal level of education (n = 3879)**				<0.001
None/Primary education	557 (14.4)	371 (19)	186 (9.6)	
Secondary education	2395 (61.7)	1251 (64.1)	1144 (59.3)	
Higher education	927 (23.9)	329 (16.9)	598 (31)	
**Maternal professional activity (n = 3849)**				<0.001
Housewives/unemployed	1433 (37.2)	835 (43)	598 (31.3)	
Training/Student	724 (18.8)	242 (12.5)	482 (25.2)	
Paid activity	1692 (44)	863 (44.5)	829 (43.4)	
**Estimated monthly income (FCFA) (n = 3734)**				<0.001
<50000	1083 (29.0)	751 (39.7)	332 (18.0)	
50000-100000	720 (19.3)	339 (17.9)	381 (20.7)	
>100000	1022 (27.4)	429 (22.7)	593 (32.2)	
Did not answer	909 (24.3)	374 (19.8)	535 (29.1)	
**Electricity at home (n = 3892)**				<0.001
No	50 (1.3)	43 (2.2)	7 (0.4)	
Yes	3792 (98.7)	1893 (97.8)	1899 (99.6)	
**Access to tap water at home (n = 3836)**				<0.001
No	1483 (38.7)	881 (45.5)	602 (31.7)	
Yes	2353 (61.3)	1054 (54.5)	1299 (68.3)	
**Presence of a functional fridge at home (n = 3823)**				<0.001
No	1450 (37.9)	915 (47.4)	535 (28.3)	
Yes	2373 (62.1)	1015 (52.6)	1358 (71.7)	
**Parity (n = 3890)**				<0.001
Primiparous	879 (22.6)	302 (15.4)	577 (29.9)	
Multiparous	3011 (77.4)	1656 (84.6)	1355 (70.1)	
**Number of antenatal visits (n = 3779)**				<0.001
<4	3324 (88.0)	1621 (85.1)	1703 (90.8)	
≥4	455 (12.0)	283 (14.9)	172 (9.2)	
**Place of delivery (n = 3913)**				<0.001
Home/Other health structure	571 (14.6)	500 (25.5)	71 (3.6)	
Pediacam study site	3342 (85.4)	1464 (74.5)	1878 (96.4)	
**Mode of delivery (n = 3908)**				0.07
Vaginal	3482 (89.1)	1768 (90.1)	1714 (88.1)	
Elective caesarian section	140 (3.6)	68 (3.5)	72 (3.7)	
Emergency caesarian section	286 (7.3)	126 (6.4)	160 (8.2)	

Adjusted on infant’s gender and recruitment site.

### Mother-infants flow after enrolment

**Figure 1:**
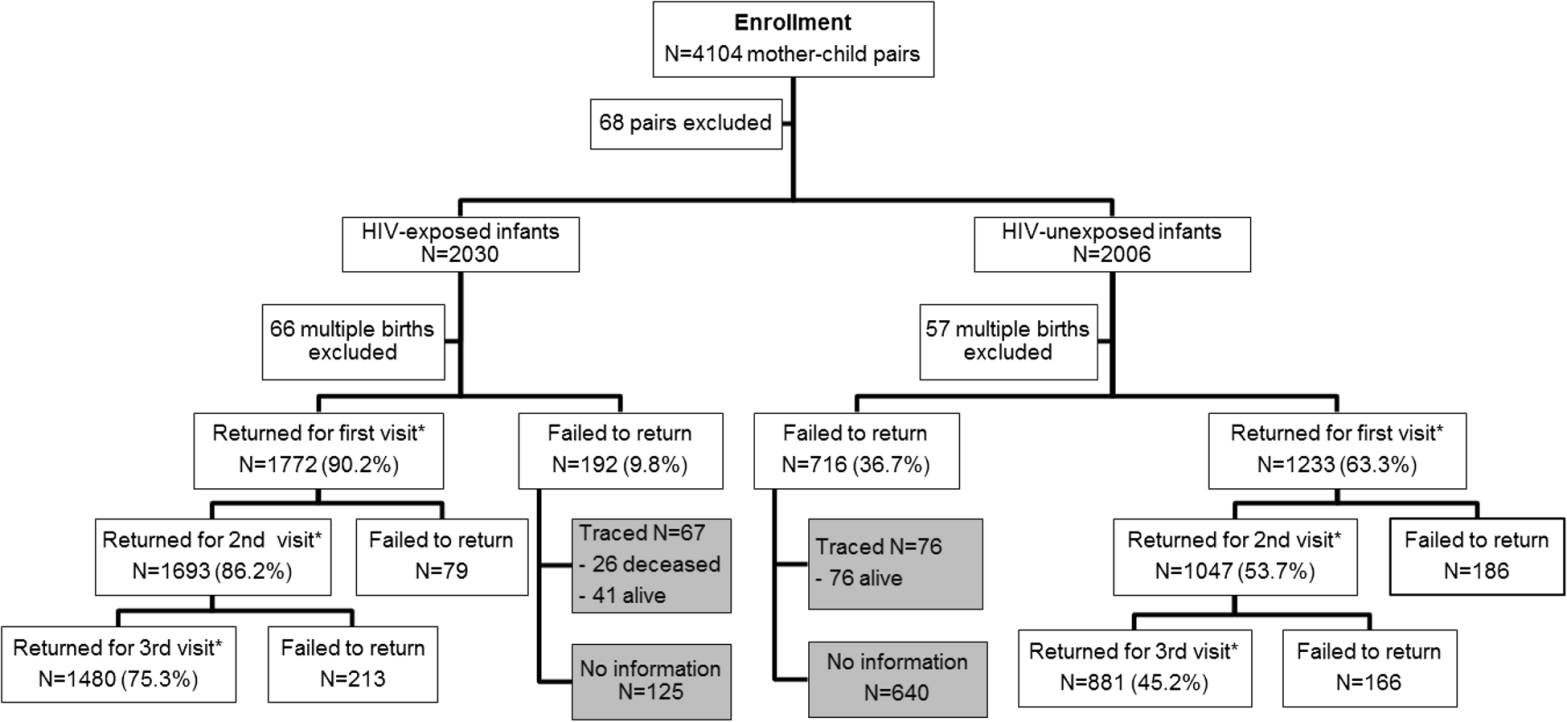
**Flow diagram of mother-child pairs from the enrolment through each follow-up visit for the ANRS 12140- Pediacam survey.** Cameroon. 2007–2010. *1st, 2nd, and 3rd visits were planned at 6, 10, 14 weeks respectively after delivery.

Figure 1 illustrates the flow of mother-child pairs from the enrolment and at each scheduled clinical visit. Overall, 23.1% (903/3913) of mother-child pairs never returned for a clinical visit and 0.1% (4/3913) only returned for visits more than 6 months after enrolment: these two groups of pairs (n = 907; 23.2%) were considered as lost to follow-up (LTFU). LTFU was significantly lower for HIV-infected mother-infant pairs than HIV-uninfected mother-infant pairs (9.8% vs 36.7%, p < .001). Among the 188 HIV-infected mother-infant pairs LTFU, only 67 could be contacted by phone. Among them, 41 infants were alive and 26 (38.8%) deceased. The median infant age at death was 4 weeks [IQR 1.7-6.1]. Seventy-six of the 715 HIV-uninfected mother-infant pairs LTFU could be contacted by phone, and all the infants were alive.

### Factors associated with LTFU

Factors related to LTFU differed between the two maternal HIV status groups. For these analyses, infants who died before the age of 6 weeks were not excluded (excluding them does not change the outcome; not shown). For HIV-infected mothers, univariable analysis indicated that the following factors were associated with LTFU: prematurity, low birth weight, infant hospitalization at birth, young maternal age (<25 years), few antenatal visits (fewer than 4), delivery by emergency caesarean section, late HIV diagnosis (during pregnancy or at delivery), absence of PMTCT prophylaxis, and TCD4 lymphocyte cell count never determined (Additional files 1, 2, 3 and 4). After adjustment, multivariable analysis (Table 2) found that infants born to mothers aged less than 25 years (aOR = 2.29, 95% CI [1.18-4.46]) and who did not take any ART prophylaxis for PMTCT (aOR = 3.45 95% CI [2.30-5.19]), and those delivered by emergency caesarean section (aOR = 2.46, 95% CI [1.47 -4.13]) were more likely to be LTFU.

**Table 2:** **Factors associated with loss-to-follow-up (LTFU, defined as never attending a clinical visit) for infants born to HIV-infected mothers, ANRS 12140-Pediacam study, Cameroon, 2007–2010: Multivariable analysis**

**Characteristics (N = 1746)**	**Lost to follow up**	
	**Adjusted OR [CI95%]**	**P value**
**Infant**		
Prematurity		
Yes (<37 weeks)	1.36 [0.87-2.15]	0.19
No (≥37 weeks)	Ref	
Low birth weight		
Yes (birth weight < 2500 grs)	1.21 [0.72-2.03]	0.48
No (birth weight ≥ 2500 grs)	Ref	
**Mother and socioeconomic status**		
Maternal age (years)		0.02
<25	2.29 [1.18-4.46]	
25-35	1.50 [0.83-2.71]	
>35	Ref	
Maternal education level		0.26
None/Primary education	1.24 [0.74-2.08]	
Secondary education	1.35 [0.93-1.95]	
Higher education	Ref	
**Antenatal care and obstetrical context of delivery**		
Delivery mode		0.005
Elective caesarian section	1.37 [0.57-3.32]	
Emergency caesarian section	2.46 [1.47-4.13]	
Vaginal	Ref	
ART prophylaxis for PMTCT		
Never	3.45 [2.3-5.19]	<0.001
Yes	Ref	

Adjusted on infant’s gender, study site, hospitalisation at birth, monthly income, number of antenatal visits, time of HIV diagnosis, CD4 cell count.

Among HIV-uninfected mother-infant pairs, being enrolled at Laquintinie hospital, young maternal age (<25 years), few antenatal visits (fewer than 4), low maternal education level, economically disadvantaged family, absence of running water at home, absence of a functional fridge at home, both lower maternal and paternal education level, and absence of both maternal and paternal socio-professional activity were associated in univariable analysis with LTFU (Additional files 1, 2, 3 and 4). Multivariable analysis (Table 3), identified maternal age < 35 years (age <25 years, aOR = 1.96, 95% CI [1.36-2.81] and 25–35 years, aOR = 1.62, 95% CI [1.15-2.29]), low maternal level of education (aOR = 2.77, 95% CI [1.95-3.95]) and mothers being housewives/unemployed (aOR = 1.56, 95% CI [1.16-2.11]), and remunerated activity of mothers (aOR = 1.52 95% CI [1.15-2.02]) as remaining independently associated with LTFU. In addition, the enrolment site, a sampling variable, was independently associated with LTFU. Mother-infant pairs enrolled at Laquintinie Hospital (LH) in Douala more likely than mother-infant pairs enrolled at the Central Hospital Maternity (CHM)/Mother and Child Center of the Chantal Biya Foundation (MCC-CBF) (aOR = 1.29, (95% CI [1.03-1.61]) to be LTFU. No significant interactions were identified between the characteristics associated with the variable of interest (LTFU) in univariable analysis. This analysis did not show differences when compared to sensitivity analysis including missing data as a category.

**Table 3:** **Factors associated with loss-to-follow-up (LTFU, defined as never attending a clinical visit) for infants born to HIV-uninfected mothers, ANRS 12140- Pediacam study, Cameroon, 2007–2010: Multivariable analysis**

**Characteristics (N = 1775)**	**Lost to follow up**	
	**Adjusted OR [CI95%]**	**P value**
**Infant**		
Recruitment site		<0.001
LH	1.29 [1.03-1.61]	
EHC	0.54 [0.41-0.69]	
MCH/MCC-CBF	Ref	
Prematurity		
Yes (<37 weeks)	1.30 [0.96-1.76]	0.37
No (≥37 weeks)	Ref	
Low birth weight		
Yes (birth weight < 2500 grs)	0.92 [0.61-1.41]	0.46
No (birth weight ≥ 2500 grs)	Ref	
**Mother and socioeconomic status**		
Maternal age (years)		<0.001
<25	1.96 [1.36-2.81]	
25-35	1.62 [1.15-2.29]	
>35	Ref	
Maternal education level		0.002
None/Primary education	2.77 [1.95-3.95]	
Secondary education	1.60 [1.28-2.00]	
Higher education	Ref	
Maternal socioprofessional activity		0.006
Housewives/Unemployed mothers	1.56 [1.16-2.02]	
Remunerated activity	1.52 [1.15-2.02]	
Training/Student	Ref	

Adjusted on infant’s gender, marital status, monthly income, access to tap water, presence of a functional fridge at home, number of antenatal visits.

## Discussion

As in most Sub-Saharan countries, Cameroon experienced a generalized HIV epidemic. The prevalence was estimated in 2011 to be 4.3% among adults aged 15–49 years with 550,000 people living with HIV and 32,800 deaths in a population of 20 million inhabitants [18]. The use of antiretroviral multitherapy has dramatically reduced mortality, transforming HIV infection from an acute to a chronic disease. One consequence is that any action intended for caring people living with HIV needs their motivation, making compliance to follow-up a key for success. We took advantage of the Pediacam cohort, launched in 2007, constituted of HIV-exposed and HIV-unexposed infants included in the first week of life and followed for five years. We used this cohort to evaluate the proportion of infants who did not return for follow-up after inclusion in the first phase of the study, and identified factors associated with this loss to follow-up (LTFU).

The overall LTFU rate was 23.2% [CI95% 22.2-24.8], and was significantly lower for HIV-infected mother-infant pairs (9.8%) than HIV-uninfected mother-infant pairs (36.7%). Few relevant studies that have addressed the problem of LTFU included a control group. In a nested case control study including 594 HIV-uninfected and 456 HIV-infected mothers in Zimbabwe [19], dropout at 9 months was 19%; the higher LTFU rate in HIV-uninfected mother-infant pairs (23.1%) is similar to our observation that LTFU among infants born to HIV-infected mothers was about 3 times higher than among infants born to HIV-uninfected mothers. The failure of HIV-uninfected mother-child pairs to return for visits might reflect their concern about stigmatization. Indeed, involving HIV-uninfected mothers in a study concerning HIV-infected mothers is a real challenge because of the possible misinterpretation that involvement indicates HIV positivity [20]. Despite the existence of information, education and communication (IEC) activities, stigma within the community is still an important reality [21]. Also, for many HIV-infected mothers, the participation in such studies is reinforced by their desire to help their infant or to facilitate their access to ART. This factor is not relevant to HIV-uninfected mothers [22]. Therefore, motivation and direct benefits associated with participation in this type of study are not equal for different groups of infants. The failure of HIV-infected mother-child pairs to return for scheduled clinical visits (9.6%) in this study was lower than reported in other African studies, including those conducted in PMTCT programmes. In Mozambique, Ethiopia, Angola, and Cameroon, LTFU rates of 75.17% [23], 47.96% [24] , 19.27% [9], and 17.55% [25], respectively, have been reported for infants by the age of 3 months. In general, LTFU tends to be lower in cohort studies than routine PMTCT programs [11]. In a cohort study conducted in South Africa, 78% of participants attended their scheduled 12-month visit whereas only 18% returned in the routine PMTCT programme [26]. The high follow-up rate in the Pediacam study may be due to the reminders by telephone, reimbursement of transport fees, provision of child care and free vaccines, and strong coordination.

The factors associated with LTFU differed according to maternal HIV status. HIV-uninfected mothers aged ≤ 35 years (<25 years and 25–35 years) more likely than those >35 years old to be LTFU. Similarly, Kaplan *et al.* [27] reported that younger patients were at greater risk of LTFU with an adjusted odds ratio of 2.2 [95% CI 1.30-3.60]. Recent evidence suggests that social factors, such as stability (income, education, occupation), are associated with LTFU [28]. In Africa, both social and economic stability increases with age. Most women aged 35 years and above are in marital relationships (89.9% of women >35 years old live with a partner versus 72% of women <25 years old). The probability of being employed is substantially greater at old than young ages (younger women are more likely to be in education. The same reasons may explain why younger mothers are more likely to be LTFU. Young participants may be geographically more mobile [29]. Lower maternal education level was also independently associated with LTFU, but only among infants born to HIV-uninfected mothers. This observation contrasts with others studies which report a higher risk of LTFU among HIV-exposed infants born to less educated mothers [30]. In India, women with graduate level education were less likely than women with less than graduate level education to be LTFU [31]. The direct benefits of HIV-study participation are not shared by HIV-uninfected mothers, their motivation to participate may be associated to their ability to understand the potential benefits of the study, such as new scientific information about HIV and its treatment; this may have a larger effect on educated participants. More than 90% of the HIV-uninfected women enrolled in this study had at least a secondary school level education.

Ioannidis *et al*. reported an association in Malawi between LTFU and teaching/student occupations: infants born to HIV-infected mothers who were teachers or students were less likely to attend follow-up visits. This might have been due to concerns about stigmatization from being identified as HIV positive. Our analysis indicated that HIV-unexposed infants born to employed mothers were more likely to be LTFU possibly because they do not have enough time off from work to attend visits. In US, Krishnan *et al.* reported that activities such as work or school prevent participants from adhering to follow-up [29].

LTFU of HIV-infected mother-infant pairs was also significantly and independently associated with the absence of ART prophylaxis. This observation is consistent with findings in Uganda presented by Ahoua *et al*. [32]; they reported a higher risk of LTFU for patients with incomplete or no ARV prophylaxis. A study in the USA reported a slightly lower LTFU rate in ARV-experienced than ARV-naïve mothers, probably due to familiarity with health-care system and a better understanding of the importance of follow-up.

We found an association between LTFU and emergency caesarean section, and this contrasts with a study in Malawi where this factor was not reported to be associated with LTFU [30]. However, our finding is similar to observations in the Pediacam study concerning factors associated to incomplete infant HIV diagnosis process. It may be because many of these women are referred from primary antenatal care settings to the hospitals participating in the Pediacam survey because of complications of labour/delivery; these women may prefer to return to their local health centres for infant follow-up after delivery [14].

A study in Malawi identified low birth weight as a significant factor influencing return to follow-up visits [30]. This was attributed to high mortality rate among low birth weight infants. In our analysis, low birth weight tended to be associated with LTFU, and was not significant in multivariable analysis. Other factors, notably hospitalization at birth and pre-term, presented the same characteristics.

In this study, 67 HIV-infected mother-infant pairs who were LTFU were traced: 26 of the infants were deceased (Figure 1).

Previous studies identified poor socio-economic conditions as a reason for LTFU. For example, HIV-infected women in India staying in families with higher economic status were found to be less likely than women living at a lower economic status to be LTFU both before and after delivery [31]. A study in South Africa identified financial difficulty as the major obstacle to attending follow-up visits for patients on antiretroviral therapy [33]. In rural Malawi, inability to afford transport costs related to the long distances to hospitals has been cited as impeding the ability to comply with protocol [21]. We did not find any such factors in our study. This divergence of results may be a consequence of the different ways that socio economic factors were assessed in these studies. In our study, we used monthly income, access to electricity at home, presence of a functional fridge at home, access to tap water at home and socio-professional activity as surrogates of economic status. We assumed that reimbursement of transport fees, and free milk, vaccines and biological exam provided by the study minimized the effects of economic status; also Pediacam operates in urban areas, where there are fewer socioeconomically disadvantaged people than in rural areas.

The factors associated with LTFU in the HIV-infected mothers group pertained to antenatal care and the obstetrical context of delivery; those associated with LTFU in the HIV-uninfected mothers group were largely relevant to socioeconomic status. For both groups of maternal HIV status, young maternal age was associated with LTFU.

One of the strengths of this study is its multicentre design including three referral hospitals with different working practices and recruitments, reflecting the diversity of practical management of PMTCT programmes. Moreover, these centres are located in two large cities of Cameroon with heterogeneous populations, albeit mostly living in urban areas. In contrast, recruitment of HIV-infected and HIV-uninfected women in the same health centres may have affected the acceptability to, and follow-up of, HIV-uninfected women, ad this is a limitation of the study. In addition, the low rate of LTFU among HIV-infected mother-child pairs, relative to other sub-Saharan Africa studies, could have reduced the power of the study to identify factors associated with LTFU.

## Conclusions

Factors associated with LTFU (never returning for a scheduled clinical visit) of mother-child pairs differ according to maternal HIV serostatus. For HIV-infected mother-child pairs, these factors pertain to the quality of antenatal care and the obstetrical context of delivery whereas for HIV-uninfected mother-child pairs, they are related to maternal characteristics and socio-economic status. Ensuring the quality of antenatal and intrapartum services could help in improving follow-up. Provision of training for clinicians in antenatal and intrapartum services may help them identify mothers at high risk of missing follow-up visits. Identification of such cases would allow targeted counselling and education about the importance of follow-up.

## Additional files

Additional file 1:
**Infant’s characteristics associated with loss-to-follow-up (LTFU, defined as never attending a clinical visit) according to maternal HIV serostatus, ANRS 12140- Pediacam study, Cameroon, 2007–2010: Univariable analysis.**
Additional file 2:
**Maternal and socio-economic characteristics associated with loss-to-follow-up (LTFU, defined as never attending a clinical visit) according to maternal HIV serostatus, ANRS 12140- Pediacam study.** Cameroon. 2007–2010: Univariable analysis. Additional file 3:
**Antenatal care and obstetrical context of delivery characteristics associated with LTFU according to maternal HIV serostatus, ANRS 12140-Pediacam survey, Cameroon, 2007–2010: Univariable analysis.**
Additional file 4:
**Paternal characteristics associated with LTFU according to maternal HIV serostatus, ANRS 12140- Pediacam study, Cameroon, 2007–2010: Univariable analysis.**
