# Supplementary material for: Different factors associated with loss to follow-up of infants born to HIV-infected or uninfected mothers: observations from the ANRS 12140-PEDIACAM study in Cameroon
Source: BMC Public Health. 2015 Mar 7;15:228. doi: 10.1186/s12889-015-1555-2 (PMC4358721; doi:10.1186/s12889-015-1555-2)
Supplement: Additional file 1: — Infant’s characteristics associated with loss-to-follow-up (LTFU, defined as never attending a clinical visit) according to maternal HIV serostatus, ANRS 12140- Pediacam study, Cameroon, 2007–2010: Univariable analysis. [file 12889_2015_1555_MOESM1_ESM.docx]

# Additional files

### Additional file 1 – Infant characteristics associated with LTFU according to maternal HIV serostatus, ANRS 12140- Pediacam study, Cameroon, 2007-2010: Univariable analysis.

|  | HIV-infected mothers | | | |  | HIV-uninfected mothers | | | |  |
| --- | --- | --- | --- | --- | --- | --- | --- | --- | --- | --- |
|  | Total | LTFU | | OR [CI95%] |  | Total | LTFU | | OR [CI95%] |  |
| Total | 1964 | 192 | % |  |  | 1949 | 716 | % |  |  |
|  |  |  |  |  |  |  |  |  |  |  |
| Recruitment site | n=1964 |  |  |  | NS | n=1949 |  |  |  | ** |
| LH | 577 | 56 | 9.7 | 1.02 (0.72-1.46) |  | 557 | 251 | 45.1 | 1.29 (1.04-1.60) |  |
| EHC | 495 | 51 | 10.3 | 1.09 (0.76-1.57) |  | 498 | 118 | 23.7 | 0.49 (0.38-0.63) |  |
| MCH/MCC-CBF | 892 | 85 | 9.5 | Ref |  | 894 | 347 | 38.8 | Ref |  |
|  |  |  |  |  |  |  |  |  |  |  |
| Gender | n=1964 |  |  |  |  | n=1949 |  |  |  |  |
| Male | 993 | 100 | 10.1 | 1.07 (0.79-1.44) | NS | 988 | 382 | 38.7 | 1.18 (0.98-1.42) | * |
| Female | 971 | 92 | 9.5 | Ref |  | 961 | 334 | 34.8 | Ref |  |
|  |  |  |  |  |  |  |  |  |  |  |
| Prematurity | n=1963 |  |  |  |  | n=1940 |  |  |  |  |
| Yes | 263 | 35 | 13.3 | 1.51 (1.02-2.23) | ** | 206 | 87 | 42.2 | 1.29 (0.96-1.73) | * |
| No | 1700 | 157 | 9.2 | Ref |  | 1734 | 627 | 36.2 | Ref |  |
|  |  |  |  |  |  |  |  |  |  |  |
| Low birth weight | n=1957 |  |  |  |  | n=1926 |  |  |  |  |
| Yes | 194 | 27 | 13.9 | 1.58 (1.02-2.44) | ** | 111 | 38 | 34.2 | 0.91 (0.61-1.36) | NS |
| No | 1763 | 164 | 9.3 | Ref |  | 1815 | 662 | 36.5 | Ref |  |
|  |  |  |  |  |  |  |  |  |  |  |
| Hospitalization at birth | n=1916 |  |  |  |  | n=1905 |  |  |  |  |
| Yes | 234 | 30 | 12.8 | 1.46 (0.96-2.22) | ** | 239 | 82 | 34.3 | 0.90 (0.67-1.19) | NS |
| No | 1682 | 154 | 9.2 | Ref |  | 1666 | 613 | 36.8 | Ref |  |
|  |  |  |  |  |  |  |  |  |  |  |
| APGAR | n=1760 |  |  |  |  | n=1780 |  |  |  |  |
| <8 | 1628 | 151 | 9.3 | 1.25 (0.71-2.2) | NS | 1671 | 600 | 35.9 | 1.21 (0.81-1.79) | NS |
| >=8 | 132 | 15 | 11.4 | Ref |  | 109 | 44 | 40.4 | Ref |  |

** Significant at p<0.05 * Significant at p<0.25 NS Non significant at p<0.05

Adjusted on infant’s gender and recruitment site except for the variables gender and recruitment site
