# Supplementary material for: Different factors associated with loss to follow-up of infants born to HIV-infected or uninfected mothers: observations from the ANRS 12140-PEDIACAM study in Cameroon
Source: BMC Public Health. 2015 Mar 7;15:228. doi: 10.1186/s12889-015-1555-2 (PMC4358721; doi:10.1186/s12889-015-1555-2)
Supplement: Additional file 2: — Maternal and socio-economic characteristics associated with loss-to-follow-up (LTFU, defined as never attending a clinical visit) according to maternal HIV serostatus, ANRS 12140- Pediacam study. Cameroon. 2007–2010: Univariable analysis. [file 12889_2015_1555_MOESM2_ESM.docx]

# Additional files

### Additional file 2 – Maternal and socio-economic characteristics associated with LTFU according to maternal HIV serostatus, ANRS 12140- Pediacam study. Cameroon. 2007-2010: Univariable analysis.

|  | HIV-infected mothers | | | |  | HIV-uninfected mothers | | | |  |
| --- | --- | --- | --- | --- | --- | --- | --- | --- | --- | --- |
|  | Total | LTFU | | OR [CI95%] |  | Total | LTFU | | OR [CI95%] |  |
| Total | 1964 | 192 | % |  |  | 1949 | 716 | % |  |  |
|  |  |  |  |  |  |  |  |  |  |  |
|  |  |  |  |  |  |  |  |  |  |  |
| Maternal age (years) | n=1963 |  |  |  | ** | n=1946 |  |  |  | ** |
| <25 | 385 | 51 | 13.2 | 1.92 (1.11-3.34) |  | 638 | 263 | 41.2 | 1.85 (1.32-2.60) |  |
| 25-35 | 1320 | 121 | 9.2 | 1.27 (0.77-2.10) |  | 1097 | 393 | 35.8 | 1.47 (1.06-2.04) |  |
| >35 | 258 | 19 | 7.4 | Ref |  | 211 | 58 | 27.5 | Ref |  |
|  |  |  |  |  |  |  |  |  |  |  |
| Marital status | n=1955 |  |  |  |  | n=1928 |  |  |  |  |
| Living alone | 604 | 60 | 9.9 | 1.03 (0.74-1.42) | NS | 569 | 193 | 33.9 | 1.17 (0.95-1.43) | * |
| Living with a partner | 1351 | 131 | 9.7 | Ref |  | 1359 | 509 | 37.5 | Ref |  |
|  |  |  |  |  |  |  |  |  |  |  |
| Maternal education level | n=1951 |  |  |  | * | n=1928 |  |  |  | ** |
| None/Primary education | 371 | 46 | 12.4 | 1.46 (0.9-2.39) |  | 186 | 98 | 52.7 | 2.97 (2.12-4.17) |  |
| Secondary education | 1251 | 116 | 9.3 | 1.06 (0.69-1.62) |  | 1144 | 443 | 38.7 | 1.69 (1.36-2.09) |  |
| Higher education | 329 | 29 | 8.8 | Ref |  | 598 | 163 | 27.3 | Ref |  |
|  |  |  |  |  |  |  |  |  |  |  |
| Maternal professional activity | n=1940 |  |  |  | NS | n=1909 |  |  |  | ** |
| Housewives / unemployed mothers | 835 | 81 | 9.7 | 0.73 (0.47-1.14) |  | 598 | 255 | 42.6 | 1.80 (1.39-2.32) |  |
| Remunerated activity | 863 | 81 | 9.4 | 0.76 (0.49-1.19) |  | 829 | 293 | 35.3 | 1.32 (1.04-1.68) |  |
| Training/School | 242 | 30 | 12.4 | Ref |  | 482 | 141 | 29.3 | Ref |  |
|  |  |  |  |  |  |  |  |  |  |  |
| Estimated Monthly income | n=1893 |  |  |  | NS | n=1841 |  |  |  | ** |
| No answer | 374 | 43 | 11.5 | 1.23 (0.78-1.93) |  | 535 | 204 | 38.1 | 1.34 (1.05-1.71) |  |
| <50000 | 751 | 74 | 9.9 | 1.03 (0.69-1.55) |  | 332 | 152 | 45.8 | 1.25 (0.95-1.64) |  |
| 50000-100000 | 339 | 28 | 8.3 | 0.85 (0.52-1.41) |  | 381 | 139 | 36.5 | 1.28 (0.98-1.67) |  |
| >100000 | 429 | 41 | 9.6 | Ref |  | 593 | 187 | 31.5 | Ref |  |
|  |  |  |  |  |  |  |  |  |  |  |
| Electricity at home | n=1936 |  |  |  |  | n=1906 |  |  |  |  |
| No | 43 | 5 | 11.6 | 1.21 (0.47-3.12) | NS | 7 | 4 | 57.1 | 2.32 (0.52-10.4) | NS |
| Yes | 1893 | 185 | 9.8 | Ref |  | 1899 | 693 | 36.5 | Ref |  |
|  |  |  |  |  |  |  |  |  |  |  |
| Access to tap water | n=1935 |  |  |  |  | n=1901 |  |  |  |  |
| No | 881 | 82 | 9.3 | 0.9 (0.66-1.22) | NS | 602 | 264 | 43.9 | 1.56 (1.28-1.90) | ** |
| Yes | 1054 | 108 | 10.2 | Ref |  | 1299 | 433 | 33.3 | Ref |  |
|  |  |  |  |  |  |  |  |  |  |  |
| Functional fridge at home | n=1930 |  |  |  |  | n=1893 |  |  |  | ** |
| No | 915 | 91 | 9.9 | 1.02 (0.76-1.38) | NS | 535 | 221 | 41.3 | 1.32 (1.08-1.62) |  |
| Yes | 1015 | 99 | 9.8 | Ref |  | 1358 | 472 | 34.8 | Ref |  |

** Significant at p<0.05 * Significant at p<0.25 NS Non significant at p<0.05

Adjusted on infant’s gender and recruitment site
