# Supplementary material for: Different factors associated with loss to follow-up of infants born to HIV-infected or uninfected mothers: observations from the ANRS 12140-PEDIACAM study in Cameroon
Source: BMC Public Health. 2015 Mar 7;15:228. doi: 10.1186/s12889-015-1555-2 (PMC4358721; doi:10.1186/s12889-015-1555-2)
Supplement: Additional file 3: — Antenatal care and obstetrical context of delivery characteristics associated with LTFU according to maternal HIV serostatus, ANRS 12140-Pediacam survey, Cameroon, 2007–2010: Univariable analysis. [file 12889_2015_1555_MOESM3_ESM.docx]

# Additional files

### Additional file 3 – Antenatal care and obstetrical context of delivery characteristics associated with LTFU according to maternal HIV serostatus, ANRS 12140-Pediacam survey, Cameroon, 2007-2010: Univariable analysis.

|  | HIV-infected mothers | | | |  | HIV-uninfected mothers | | | |  |
| --- | --- | --- | --- | --- | --- | --- | --- | --- | --- | --- |
|  | Total | LTFU | | OR [CI95%] |  | Total | LTFU | | OR [CI95%] |  |
| Total | 1964 | 192 | % |  |  | 1949 | 716 | % |  |  |
|  |  |  |  |  |  |  |  |  |  |  |
|  |  |  |  |  |  |  |  |  |  |  |
| Primigravid | n=1958 |  |  |  |  | n=1932 |  |  |  |  |
| Yes | 302 | 32 | 10.6 | 1.11 (0.74-1.65) | NS | 577 | 212 | 36.7 | 1.02 (0.83-1.24) | NS |
| No | 1656 | 160 | 9.7 | Ref |  | 1355 | 493 | 36.4 | Ref |  |
|  |  |  |  |  |  |  |  |  |  |  |
| Number of antenatal visits | n=1904 |  |  |  |  | n=1875 |  |  |  |  |
| <4 | 1621 | 147 | 9.1 | 1.56 (1.06-2.28) | ** | 1703 | 609 | 35.8 | 1.64 (1.19-2.24) | ** |
| >4 | 283 | 38 | 13.4 | Ref |  | 172 | 82 | 47.7 | Ref |  |
|  |  |  |  |  |  |  |  |  |  |  |
| Mode of delivery | n=1962 |  |  |  | ** | n=1946 |  |  |  | NS |
| Elective caesarean section | 68 | 8 | 11.8 | 1.35 (0.63-2.87) |  | 72 | 30 | 41.7 | 1.26 (0.78-2.04) |  |
| Emergency caesarean sec | 126 | 25 | 19.8 | 2.50 (1.57-4.00) |  | 160 | 65 | 40.6 | 1.21 (0.87-1.68) |  |
| Vaginal | 1768 | 159 | 9.0 | Ref |  | 1714 | 619 | 36.1 | Ref |  |
|  |  |  |  |  |  |  |  |  |  |  |
| Place of delivery | n=1964 |  |  |  |  | n=1949 |  |  |  |  |
| Home/Other health center | 500 | 50 | 10.0 | 1.03 (0.74-1.45) | NS | 71 | 28 | 39.4 | 1.13 (0.69-1.83) | NS |
| Pediacam study site | 1464 | 142 | 9.7 | Ref |  | 1878 | 688 | 36.6 | Ref |  |
|  |  |  |  |  |  |  |  |  |  |  |
| Time of HIV diagnosis | n=1964 |  |  |  |  | / | / | / | / | / |
| Pregnancy/Delivery | 1087 | 129 | 11.9 | 1.74 (1.27-2.39) | ** | / | / | / | / | / |
| Before pregnancy | 877 | 63 | 7.2 | Ref |  | / | / | / | / | / |
|  |  |  |  |  |  |  |  |  |  |  |
| HIV status disclosure | n=1823 |  |  |  | * | n=1654 |  |  |  | NS |
| Don’t know | 28 | 6 | 21.4 | 2.17 (0.83-5.7) |  | 26 | 12 | 46.2 | 1.97 (0.68-5.75) |  |
| Yes | 1454 | 132 | 9.1 | 0.8 (0.54-1.17) |  | 1595 | 588 | 36.9 | 1.34 (0.63-2.84) |  |
| No | 341 | 38 | 11.1 | Ref |  | 33 | 10 | 30.3 | Ref |  |
|  |  |  |  |  |  |  |  |  |  |  |
| CD4 cell count | n=1860 |  |  |  |  | / | / | / | / | / |
| Never done | 306 | 58 | 19.0 | 2.77 (1.97-3.89) | ** | / | / | / | / | / |
| Done | 1554 | 121 | 7.8 | Ref |  | / | / | / | / | / |
|  |  |  |  |  |  |  |  |  |  |  |
| ART prophylaxis for PMTCT | n=1162 |  |  |  |  | / | / | / | / | / |
| Never | 712 | 76 | 10.7 | 3.66 (2.52-5.3) | ** | / | / | / | / | / |
| Yes | 450 | 30 | 6.7 | Ref |  | / | / | / | / | / |
|  |  |  |  |  |  |  |  |  |  |  |

** Significant at p<0.05 * Significant at p<0.25 NS Non significant at p<0.05

Adjusted on infant’s gender and recruitment site

### Additional file 4 – Paternal characteristics associated with LTFU according to maternal HIV serostatus, ANRS 12140-Pediacam study, Cameroon, 2007-2010: Univariate analysis.

|  | HIV-infected mothers | | | |  | HIV-uninfected mothers | | | |  |
| --- | --- | --- | --- | --- | --- | --- | --- | --- | --- | --- |
|  | Total | LTFU | | OR [CI95%] |  | Total | LTFU | | OR [CI95%] |  |
| Total | 1964 | 192 | % |  |  | 1949 | 716 | % |  |  |
|  |  |  |  |  |  |  |  |  |  |  |
|  |  |  |  |  |  |  |  |  |  |  |
| Paternal age (years) |  |  |  |  |  |  |  |  |  |  |
| <35 | 696 | 76 | 10.9 | 1.27 (0.93,1.75) | * | 907 | 320 | 35.3 | 1.01 (0.83-1.23) | NS |
| >=35 | 1093 | 96 | 8.8 | Ref |  | 829 | 290 | 35.0 | Ref |  |
|  |  |  |  |  |  |  |  |  |  |  |
| Paternal education level |  |  |  |  | NS |  |  |  |  | ** |
| None/Primary education | 454 | 43 | 9.5 | 0.89 (0.59,1.36) |  | 324 | 141 | 43.5 | 1.73 (1.32-2.27) |  |
| Secondary education | 797 | 77 | 9.7 | 0.91 (0.63,1.32) |  | 685 | 269 | 39.3 | 1.46 (1.17-1.81) |  |
| Higher education | 515 | 54 | 10.5 | Ref |  | 738 | 227 | 30.8 | Ref |  |
|  |  |  |  |  |  |  |  |  |  |  |
| Paternal professional activity |  |  |  |  | NS |  |  |  |  | ** |
| Training/School | 64 | 10 | 15.6 | 1.87 (0.70,5.05) |  | 126 | 36 | 28.6 | 0.38 (0.20-0.75) |  |
| Remunerated activity | 1679 | 161 | 9.6 | 1.07 (0.51,2.26) |  | 1624 | 581 | 35.8 | 0.54 (0.31-0.94) |  |
| Unemployed | 89 | 8 | 9.0 | Ref |  | 51 | 26 | 51.0 | Ref |  |
|  |  |  |  |  |  |  |  |  |  |  |

** Significant at p<0.05 * Significant at p<0.25 NS Non significant at p<0.05
