# Supplementary material for: Different factors associated with loss to follow-up of infants born to HIV-infected or uninfected mothers: observations from the ANRS 12140-PEDIACAM study in Cameroon
Source: BMC Public Health. 2015 Mar 7;15:228. doi: 10.1186/s12889-015-1555-2 (PMC4358721; doi:10.1186/s12889-015-1555-2)
Supplement: Additional file 4: — Paternal characteristics associated with LTFU according to maternal HIV serostatus, ANRS 12140- Pediacam study, Cameroon, 2007–2010: Univariable analysis. [file 12889_2015_1555_MOESM4_ESM.docx]

# Additional files

### Additional file 4 – Paternal characteristics associated with LTFU according to maternal HIV serostatus, ANRS 12140-Pediacam study, Cameroon, 2007-2010: Univariable analysis.

|  | HIV-infected mothers | | | |  | HIV-uninfected mothers | | | |  |
| --- | --- | --- | --- | --- | --- | --- | --- | --- | --- | --- |
|  | Total | LTFU | | OR [CI95%] |  | Total | LTFU | | OR [CI95%] |  |
| Total | 1964 | 192 | % |  |  | 1949 | 716 | % |  |  |
|  |  |  |  |  |  |  |  |  |  |  |
|  |  |  |  |  |  |  |  |  |  |  |
| Paternal age (years) | n=1789 |  |  |  |  | n=1736 |  |  |  |  |
| <35 | 696 | 76 | 10.9 | 1.27 (0.93,1.75) | * | 907 | 320 | 35.3 | 1.01 (0.83-1.23) | NS |
| >35 | 1093 | 96 | 8.8 | Ref |  | 829 | 290 | 35.0 | Ref |  |
|  |  |  |  |  |  |  |  |  |  |  |
| Paternal education level | n=1766 |  |  |  | NS | n=1747 |  |  |  | ** |
| None/Primary education | 454 | 43 | 9.5 | 0.89 (0.59,1.36) |  | 324 | 141 | 43.5 | 1.73 (1.32-2.27) |  |
| Secondary education | 797 | 77 | 9.7 | 0.91 (0.63,1.32) |  | 685 | 269 | 39.3 | 1.46 (1.17-1.81) |  |
| Higher education | 515 | 54 | 10.5 | Ref |  | 738 | 227 | 30.8 | Ref |  |
|  |  |  |  |  |  |  |  |  |  |  |
| Paternal professional activity | n=1832 |  |  |  | NS | n=1801 |  |  |  | ** |
| Training/School | 64 | 10 | 15.6 | 1.87 (0.70,5.05) |  | 126 | 36 | 28.6 | 0.38 (0.20-0.75) |  |
| Remunerated activity | 1679 | 161 | 9.6 | 1.07 (0.51,2.26) |  | 1624 | 581 | 35.8 | 0.54 (0.31-0.94) |  |
| Unemployed | 89 | 8 | 9.0 | Ref |  | 51 | 26 | 51.0 | Ref |  |
|  |  |  |  |  |  |  |  |  |  |  |

** Significant at p<0.05 * Significant at p<0.25 NS Non significant at p<0.05

Adjusted on infant’s gender and recruitment site
